# Enhancing Dental Students’ Understanding of Behavior Management in Pediatric Dentistry: A Comparison of Two Teaching Methods

**DOI:** 10.7759/cureus.25342

**Published:** 2022-05-25

**Authors:** Monika Khubchandani, Tripti Srivastava, Nilima R Thosar

**Affiliations:** 1 Pediatric Dentistry, Sharad Pawar Dental College and Hospital, Datta Meghe Institute of Medical Sciences, Wardha, IND; 2 Physiology, Jawaharlal Nehru Medical College and Hospital, Datta Meghe Institute of Medical Sciences, Wardha, IND; 3 Pediatric and Preventive Dentistry, Sharad Pawar Dental College and Hospital, Datta Meghe Institute of Medical Sciences, Wardha, IND

**Keywords:** pediatric dentistry, dental students, role play, group discussion, behavior management

## Abstract

Aim

Communication, behavioral, and attitudinal skills are an integral part of teaching behavior management to dental undergraduate students. Due to the complexity of these skills, clinical teaching through the didactic method imparts minimal capacity for adopting a deep approach to learning. The implications of role play and group discussion could be an opportunity to facilitate such learning outcomes. The objectives of this study were to evaluate and compare the efficacy of role play and group discussion as teaching-learning methods for behavior management in pediatric dentistry.

Material and methods

The study was carried out with 92 final-year Bachelor of Dental Surgery (BDS) undergraduate students at Sharad Pawar Dental College and Hospital, Datta Meghe Institute of Medical Sciences, Wardha, India. The Tell-Show-Do and non-pharmacological behavior management techniques were taught to the intervention and control groups by the role-play and group discussion methods, respectively. To assess knowledge gain, a pre-test and post-test were conducted. To evaluate the acquisition of communication, behavioral, and attitudinal skills, students were made to perform a clinical procedure, i.e. placement of pit and fissure sealant in primary molars. Each student was evaluated by direct observation using a checklist.

Results

The results revealed a statistically significant difference between the post-test scores of the role-play and group discussion methods, with average values of 8.57±0.98 and 6.97±0.12, respectively (*p=* 0.020). The average scores of communication and attitudinal skills among the intervention and control groups were 19.70±0.87 and 13.98±1.51, respectively (*p*=0.027). Hence, the role-play method was found to be a highly effective method.

Conclusion

Role-play as a teaching tool was highly effective in instilling behavior management skills among students to deal with young patients in clinical situations as compared to the group discussion method. The difference is depicted in higher scores in the intervention group.

## Introduction

Behavior management is considered to be the key element in the management of children in pediatric dentistry [1]. McElroy (1895) defined behavior management as “Although the operative dentistry may be perfect, the appointment is a failure if the child departs in tears.” This was the first criteria mentioning the evaluation of the success or failure of a child’s appointment in the dental operatory [2]. Since childhood experience plays an important role in forming adult behavior, proper behavior management from the early stages will help in the development of a positive oral health attitude among individuals throughout life [3].

Traditionally, behavior management is taught to dental students during lectures and through group discussions. In both the teaching methods, students receive directions to deal with young patients during clinical situations. This form of transmission of knowledge may be useful for rote learning, but this method is ineffective at providing students with the necessary clinical skills required for patient management in the dental operatory [4-5].

Since effective communication with the child is the backbone of all behavior management techniques, role-play is perceived as gratifying in terms of developing effective communication and appropriate behavioral and attitudinal skills [6]. In role-play, the learner develops and practices newly acquired skills by simulating a scenario. It involves a minimum of two or a group of students, who communicate both as dentists and as patients as they switch between these roles [7-8].

Students in the health care profession need a higher level of critical thinking to develop patient management in terms of communication, attitude, behavior, and clinical judgment skills. A small group discussion is one method that creates an active learning environment [9]. However, there are no published reports that can highlight the use of role-playing as a teaching tool in dental education. Hence, the present study was undertaken with the intention to study the effectiveness of role-playing in comparison to group discussions as a teaching-learning method for behavior management in pediatric dental patients.

## Materials and methods

The present study was conducted at Sharad Pawar Dental College and Hospital, Datta Meghe Institute of Medical Sciences, Wardha, for the duration of one year after receiving ethical approval from the institutional ethics committee (DMIMS (D.U.)/IEC/2017-18/6269). The participants included 92 final-year Bachelor of Dental Surgery (BDS) students who were willing to voluntarily participate in the study. Written informed consent was taken from each participant prior to the start of the study. All 92 study participants were sensitized to the role-play as well as group discussion methods of teaching and learning.

Sensitization of students to role-play

A mini-workshop was conducted for sensitizing students to the role-play method. The students were explained how to design the role-play settings, the objectives of the role-play activity, and its relevance in professional education. Roleplay videos were also used to explain the role-playing process.

Sensitization of students to group discussions

A second workshop was conducted by the investigator for sensitizing students to the group discussion method of teaching and learning.

Random assignment 

Following sensitization, students were randomly assigned to Group A (Role-Play) and Group B (Group Discussion) using the lottery method. On the basis of a review of the guidelines on behavior guidance, a questionnaire to test knowledge gain and a skill-based assessment tool to test the communication and attitudinal skills were designed.

Actual conduct of role-playing and group discussions

The topic selected for the study was Tell-Show-Do and non-pharmacological behavior management. Following sensitization, students were divided into two groups by simple random sampling. A pre-test for both groups was conducted. A questionnaire including 10 multiple-choice questions was distributed among students. Students of group A were divided into five subgroups. The role-play method included the principal investigator and eight students of each subgroup. The role play proceeded for about 20-25 minutes where the principal investigator focused on certain points like the use of nonverbal communication skills, implementation of the Tell-Show-Do technique during treatment, and positive reinforcement of patient toward dental care.

**Figure 1:**
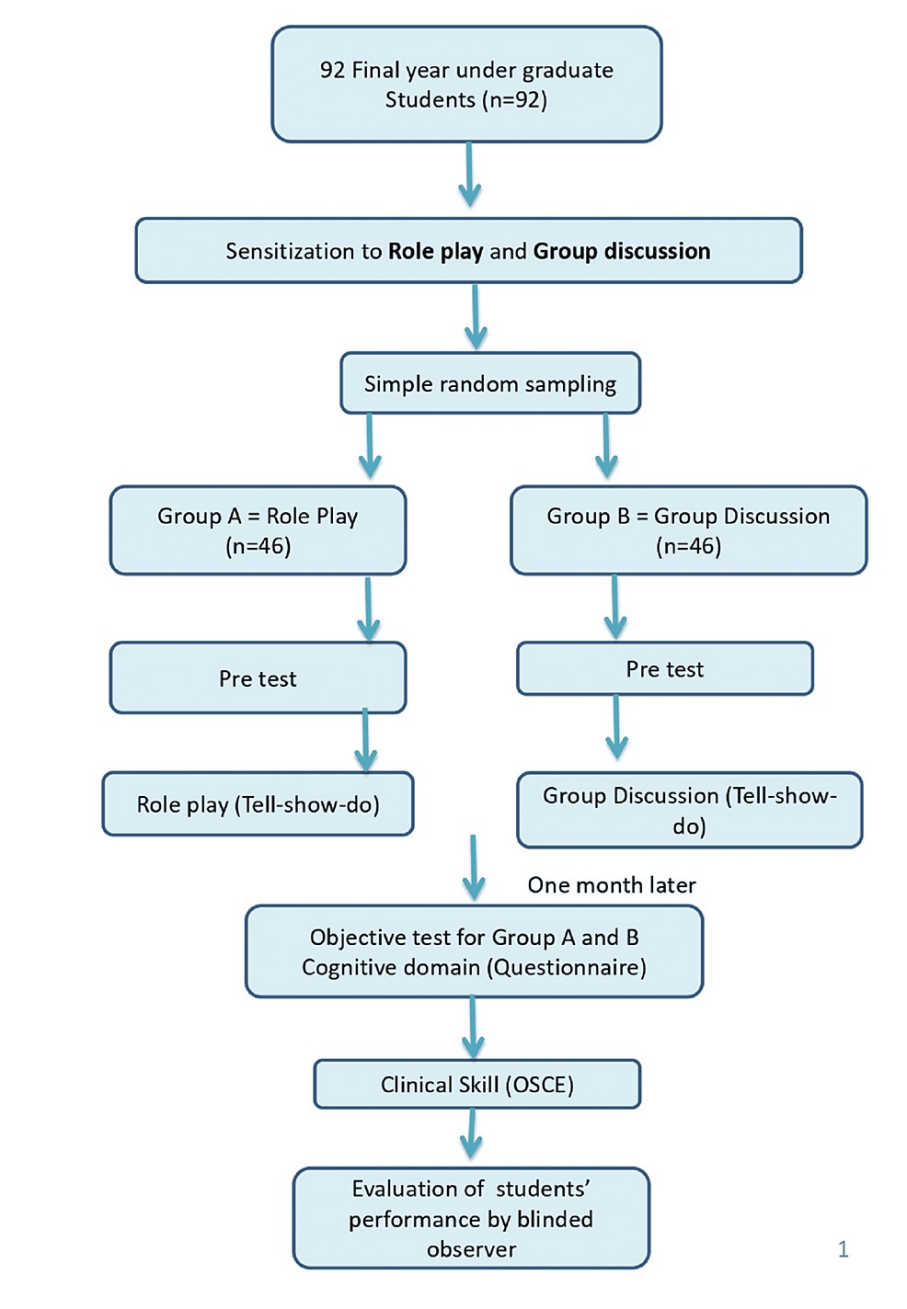
Flow chart showing the study protocol

Students of Group B were also divided into five subgroups. The small group discussion was conducted with each subgroup for 30 minutes where the principal investigator explained the topic in detail with all the aspects covered in role play. The students were also given the opportunity to participate in discussions and ask questions. To test retention of the topic, a surprise objective test of 10 questions, and to assess the communication and attitudinal skills, an objective structured clinical examination (OSCE) test were conducted after one month. In the OSCE test, students of both the groups were made to perform a clinical procedure (placement of pit and fissure sealant) in three-four-year-old patients with fully erupted primary molars having a deep fissure. Children with past dental experience were excluded from the study. Other exclusion criteria were: teeth with carious lesions, children with medical conditions, presence of hypoplastic teeth, and children for whom parents were unable to give consent. Sealant placement was carried out during the second dental visit of each patient. Each student was taken to a well-equipped special operating room where he/she engaged with a patient for about 15 minutes and performed the clinical procedure. An evaluator, who was blinded to the study design and the students’ group assignment, evaluated students' performance using a checklist. The crossover was done after a washout period of three months to minimize bias in the study. The perception of participants of both groups regarding two teaching methods was obtained. The study protocol is depicted in Figure 1.

Data analysis

For assessing the effectiveness of the role-play and group discussion methods, a descriptive inferential analysis was performed. The mean scores of both groups were compared by the unpaired t-test. The difference between scores was considered significant if the p-value was less than 0.05.

## Results

A statistically significant difference was observed between the mean pre-test and post-test scores of Group A students subjected to the role-play method and Group B students subjected to the group discussion method. When the pre-test scores of the intervention group (role-play) and the control group (group discussion) were compared, the average scores were 2.48±0.78 and 2.64±0.62, respectively (Table 1).

**Table 1:** Comparison of pretest score (cognitive domain)

Group	n	Mean	Std. Deviation	Std. Error Mean	p-value
Role-play	46	2.48	0.78	0.12	0.088
Group discussion	46	2.64	0.62	0.09	

When comparing post-test scores, a statistically significant difference was found, with average scores of 8.57±0.98 and 6.97±0.12 in the intervention and control groups, respectively (p<0.05) (Table 2). These findings suggest that learning behavior management with role-play was highly effective as compared to the group discussion method.

**Table 2:** Comparison of the post-test scores (cognitive domain)

Group	n	Mean	Std. Deviation	Std. Error Mean	p-value
Role Play	46	8.57	0.98	0.14	0.020
Group Discussion	46	6.97	0.12	0.04	

The average pre-test and post-test scores were 2.48±0.78 and 8.57±0.98 in Group A (role-play) while they were 2.64±0.62 and 6.97±0.12 in Group B (group discussion), respectively, showing a statistically significant difference (p<0.05) (Tables 3-4).

**Table 3:** Comparison of cognitive pre and post-test groups in role play

Group	n	Mean	Std. Deviation	Std. Error Mean	p-value
Pre-test	46	2.48	0.78	0.12	0.001*
Post-test	46	8.57	0.98	0.14	

**Table 4:** Comparison of cognitive pre and post-test scores in group discussion

Group	n	Mean	Std. Deviation	Std. Error Mean	p-value
Pre-test	46	2.64	0.62	0.09	0.001*
Post-test	46	6.97	0.12	0.04	

The overall clinical competence, including communication skills, attitudinal skills, and the implementation of the Tell-Show-Do method in both groups was assessed by direct observation using a checklist. When the data were subjected to the unpaired t-test, a statistically significant difference was obtained between the two groups. The mean scores in Group A and Group B were 19.70±0.87 and 13.98±1.51, respectively (p<0.05) (Table 5). This implies that students who had been taught/instructed with the Tell-Show-Do technique by a group discussion performed notably less than those who observed the faculty employing this management approach in clinical practice.

**Table 5:** Comparison of overall clinical competence (communication, behavioral, and attitudinal skills)

Group	N	Mean	Std. Deviation	Std. Error Mean	p-value
Role Play	46	19.70	0.87	0.13	0.027
Group Discussion	46	13.98	1.51	0.22	

The perception of participants of both groups regarding two teaching methods with reference to learning and development of required competencies revealed noticeable differences as depicted in Table 6.

**Table 6:** Perception of students of the role-play and group discussion methods with reference to the learning and development of competencies

Items	Number of students who agreed in role-play (RP) (n=46)	Number of students who agreed in group discussion (n=46)
Learning becomes easier in RP	72 (78%)	20 (21%)
Learned better communication skills in RP	78 (85%)	14 (15%)
RP offers the opportunity for active participation	79 (86 %/)	13 (14%)
RP help students to develop required competencies (knowledge, attitude, and skills)	77 (83%)	15 (17%)

.

## Discussion

Research on the use of role-play has largely been used to acquire comprehensive expertise, including knowledge, skill, problem-solving ability, attitude, and so on. But, based on a systematic literature search, it has been found that the potential of role-playing is not being used as a tool to impart subject knowledge in dental education.

In the present study, sensitization to group discussion was conducted for both groups. Along with this, a second training workshop about role-play for both groups was conducted. This was in accordance with Wong ML et al. who organized a training session for students at the beginning of the role-play where they had hands-on practice in applying the communication skills [10]. Similarly, in the study by Maha et al., prior to teaching generic skills, delegates were required to attend training sessions to develop communication and presentation skills [11].

A significant difference was observed in the mean scores of knowledge gain by students in the intervention (8.57±0.98) and control groups (6.97±0.12) (p<0.05) (Table 2). The findings suggest that even though considerable learning occurred in both groups, the role-play method was more advantageous. Although no studies have been conducted to compare role-play and group discussion in teaching behavior management, in agreement with our findings, Kristina M et al. measured knowledge retention in terms of subjective reports and objective assessment at two distinct times and reported role-play simulation as an effective teaching technique [12]. Likewise, Hober C et al. found role-play to be effective when they analyzed nursing students’ perceptions of role-playing in high-fidelity simulation [13].

Overall clinical competence, including communication and attitudinal skills and implementation of the Tell-Show-Do behavior management method in both groups, was assessed by direct observation using a checklist. The mean scores in the intervention and control groups were 19.70±0.87 and 13.98±1.51, respectively (p<0.05) (Table 3).

This difference in the overall clinical competence of students can be attributed to the learner-centered active nature of the role-play method, which incorporates simulations, games, and demonstrations of real-life cases that can engage learners [14].

In the present study, statistically significant differences (p<0.05) were obtained when the knowledge gain and overall clinical competence of the two groups were compared. This could be attributed to the very nature of the role-play technique that allows students to explore realistic situations by interacting with other people in contrast to group discussions where students simply take part in interactive sessions supervised by a facilitator. Furthermore, group discussions have a tendency to benefit the stronger students - the individuals already more familiar with the subject [15].

In the current research, a majority of the students perceived that role-play helps develop comprehensive competencies, including knowledge, skills, and attitude. Similar findings were reported by D'cruz SM, who stated that the role-play method is interesting, lively, and helpful in breaking monotony [16].

Limitations

The limitation of the present study is that it is based on subjects obtained from a single year of dental students at one institute. Due to feasibility constraints, the baseline data of knowledge of the concerned topic and the communication and attitudinal skills of dental students while dealing with young patients in the clinical setting were not recorded. Also, only one experience during the clinical posting was tested for a limited period of time. Hence, further research in this field is required in the form of extensive long-term studies.

## Conclusions

In our study, considerable learning occurred in both methods. However, role-play certainly was found to be more effective for dental undergraduates when knowledge gain and acquisition of communication and attitudinal skills were considered. Hence, role-play can be considered a valuable teaching-learning tool for dental students. Role-play can be recommended by educators as a new teaching modality, as it offers the opportunity for active student engagement and the integration of learned concepts into practice.

## References

[1] Roberts JF, Curzon ME, Koch G, Martens LC (2010). Review: behaviour management techniques in paediatric dentistry. Eur Arch Paediatr Dent.

[2] Wright GZ, Kupietzky A (2014). Behavior management in dentistry for children. Iowa USA, Wiley-Blackwell.

[3] Adair SM (2004). A survey of members of the American Academy of Pediatric Dentistry on their use of behavior management techniques. Pediatr Dent.

[4] Hafezimoghadam Hafezimoghadam (2013). A comparative study of lecture and discussion methods in the education of basic life support and advanced cardiovascular life support for medical students. Tr J Emerg Med.

[5] Amin Z, Eng KH (2003). Basics in Medical Education. Ltd.

[6] Alkin MC, Christie CA (2002). The use of role-play in teaching evaluation. Am J Eval.

[7] Chesler M, Fox R (1966). Role-Playing Methods in the Classroom. https://eric.ed.gov/?id=ED075276.

[8] Tufford L, Bogo M, Asakura K (2018). Simulation versus role-play: perception of prepracticum BSW students. Journal of Baccalaureate Social Work.

[9] Arias A, Scott R, Peters OA, McClain E, Gluskin AH (2016). Educational outcomes of small group discussion versus traditional lecture format in dental students’ learning and skills acquisition. J Dent Res.

[10] Wong ML, Peng L (2016). Using role play and standardized patients in pre-clinical communication training: attitudes and perceptions of dental undergraduates. Asian Journal of the Scholarship of Teaching and Learning.

[11] Maha MA (2014). Using peer-assisted learning and role-playing to teach generic skills to dental students: the health care simulation model. J Dent Res.

[12] DeNeve KM, Heppner MJ (1997). Role play simulations: the assessment of an active learning technique and comparisons with traditional lectures. Innov High Educ.

[13] Hober C, Bonnel W (2014). Student perceptions of the observer role in high-fidelity simulation. Clin Simul Nurs.

[14] Rashid S, Qaisar S (2017). Role play: a productive teaching strategy to promote critical thinking. Bull Educ Res.

[15] Larson BE (2000). Classroom discussion: a method of instruction and a curriculum outcome. Teach Teach Educ.

[16] D'cruz SM, Muthukumar S, Rajaratnam N, Anandarajan B (2013). Perception of medical students in India about the use of role-play as a teaching-learning method in physiology. Int J Biomed Adv Res.

